# The Role of the CXCL12/CXCR4/ACKR3 Axis in Autoimmune Diseases

**DOI:** 10.3389/fendo.2019.00585

**Published:** 2019-08-27

**Authors:** Eva M. García-Cuesta, César A. Santiago, Jesús Vallejo-Díaz, Yasmina Juarranz, José Miguel Rodríguez-Frade, Mario Mellado

**Affiliations:** ^1^Department of Immunology and Oncology, Centro Nacional de Biotecnología/CSIC, Madrid, Spain; ^2^Macromolecular X-Ray Crystallography Unit, Centro Nacional de Biotecnología/CSIC, Madrid, Spain; ^3^Department Cell Biology, Research Institute Hospital 12 de Octubre (i+12), Complutense University of Madrid, Madrid, Spain

**Keywords:** chemokines/chemokine receptors, inflammation, autoimmunity, CXCL12 chemokine, CXCR4 = chemokine receptor 4, ACKR3

## Abstract

Chemokine receptors are members of the G protein-coupled receptor superfamily. These receptors are intimately involved in cell movement, and thus play a critical role in several physiological and pathological situations that require the precise regulation of cell positioning. CXCR4 is one of the most studied chemokine receptors and is involved in many functions beyond leukocyte recruitment. During embryogenesis, it plays essential roles in vascular development, hematopoiesis, cardiogenesis, and nervous system organization. It has been also implicated in tumor progression and autoimmune diseases and, together with CD4, is one of the co-receptors used by the HIV-1 virus to infect immune cells. In contrast to other chemokine receptors that are characterized by ligand promiscuity, CXCR4 has a unique ligand—stromal cell-derived factor-1 (SDF1, CXCL12). However, this ligand also binds ACKR3, an atypical chemokine receptor that modulates CXCR4 functions and is overexpressed in multiple cancer types. The CXCL12/CXCR4/ACKR3 axis constitutes a potential therapeutic target for a wide variety of inflammatory diseases, not only by interfering with cell migration but also by modulating immune responses. Thus far, only one antagonist directed against the ligand-binding site of CXCR4, AMD3100, has demonstrated clinical relevance. Here, we review the role of this ligand and its receptors in different autoimmune diseases.

## Introduction

The human chemokine family is defined by almost 50 low-molecular-weight proteins, originally described as pro-inflammatory cytokines (1). By associating with and signaling through seven transmembrane G protein-coupled receptors (GPCRs), they activate multiple signaling pathways in target cells. Chemokines are essential for the spatio-temporal organization of leukocytes (2), and thereby ensure the correct organization and function of the immune system. Not surprisingly, dysregulation of chemokine production often results in disease. The chemokine system is complex and, in some cases, even redundant, with several chemokines that bind the same receptor with similar affinities and receptors that can bind the same ligand. Moreover, cells can express simultaneously or during different stages of their life several receptors at the cell membrane (3–7). This apparent promiscuity is, nonetheless, contentious and raises questions about the relevance of one or multiple chemokines or chemokine receptors in a determined pathological or physiological process. For example, through binding to CCR7, CCL19, and CCL21 are implicated in lymph node homeostasis and regulate naïve T cell encounters with mature antigen-presenting cells (8, 9). It is known, however, that both ligands promote differential G protein-coupled receptor kinase (GRK) recruitment to CCR7 and, in consequence, differentially activate β-arrestins (10, 11). These chemokines display differential interaction—and docking—modes for CCR7 leading to stabilization of different receptor conformations and hereby preferential activation of distinct intracellular signaling pathways (12).

Despite the crucial role of chemokines in immune function, efforts, and resources invested in the development of specific and efficient drugs have yielded poor results. Indeed, only two drugs targeting chemokine receptors have been approved for clinical use so far, and they apply to very specific situations: HIV-1 infection in the case of the CCR5 antagonist maraviroc (13) and stem cell mobilization from the bone marrow (BM) in the case of the CXCR4 antagonist AMD3100 (plerixafor) (14).

New findings have demonstrated that the chemokine/chemokine receptor system is even more complex. For instance, chemokines are capable of forming dimers and oligomers (15–17), and can interact with synergy-inducing molecules (18) and with glycosaminoglycans (GAGs) (17, 19, 20). It is also known that chemokine receptors are not only monomeric entities, but also exist as dimers and higher-order oligomers (21) that also interact with other membrane proteins (21–24) and exhibit signaling crosstalk with other proteins (25, 26).

A good example of the complex biology of chemokine signaling is the ubiquitously expressed CXCL12, which signals by binding to two receptors, CXCR4 and ACKR3. The CXCL12/CXCR4/ACKR3 axis plays key roles in many physiological and pathological processes, including embryogenesis, wound healing processes, angiogenesis, in the development and metastasis of tumors and during HIV-1 infection. The available evidence indicates that this signaling axis is also essential in maintenance of homesostasis and for host defense, and participates in progression of inflammation. The present review discusses the role of the CXCL12/CXCR4/ACKR3 axis in inflammation, focusing on its involvement in several autoimmune diseases.

## CXCL12, the Ligand

Initially known as stromal cell-derived factor-1α (SDF-1 α) or preB-cell growth-stimulating factor (PBSF), CXCL12 is probably the most studied member of the chemokine family (27, 28). It is a homeostatic chemokine produced in multiple tissues including lymph nodes (LNs), brain, liver, colon, kidney, testis, lung, pancreas, skin and placenta, and in different cell types including stromal cells, osteoblasts, fibroblasts, dendritic cells and monocytes, among others. Data from *Cxcl12*-deficient mice, corroborated by those obtained in mice lacking the gene for its receptor, CXCR4, reveal its essential role in physiology. Both mouse models are embryonic lethal with severe defects in hematopoiesis and in the nervous and cardiovascular systems (29–31). In adults, CXCR4/CXCL12 participates in the retention of hematopoietic stem cells in BM, in the traffic of T cell-precursors to the thymus, and in the clearance of neutrophils in the BM. In cooperation with CCR7 ligands, CXCL12 participates in T lymphocyte homing, it assists lymphocyte trafficking across the high endothelial venules into LNs and Peyer's patches (32). CXCL12 directs T central memory cell homing to LNs (33). Interestingly, a role for CXCL12 in determining the location of metastasis in different tumors has been reported (34), and several studies point to its potential as a biomarker (35) in hepatocellular carcinoma (36), bladder cancer (37) and glioma recurrence (38), and as a predictor of poor survival in ovarian cancer (39). CXCL12 also has prognosis potential in non-tumoral processes such as cirrhosis (40), diabetes (41), and cardiovascular diseases (42).

CXCL12 also interacts with GAGs through a cluster of basic residues—the BBXB motif (43). The interaction with GAGs is necessary for the *in vivo* activity of certain chemokines (17), contributing to the complexity of the system (19). It increases the local concentration of chemokines, presents the ligand to the receptors, and allows the formation of chemokine gradients (17). The existence of partial overlap between GAG and receptor binding sites on CXCL12 suggests that chemokine oligomerization may allow simultaneous binding (15). However, recent data suggest that binding to CXCR4 competes with CXCL12 dimerization, which argues against GAG-mediated presentation (20). Although CXCR4 was initially described as the unique receptor for CXCL12, CXCL12 also binds the atypical receptor ACKR3, also known as CXCR7 (44). This receptor does not activate G proteins, but interacts with β-arrestins (45), indicating that it is likely to be more than just a scavenger receptor for CXCL12.

## CXCR4/ACKR3, the Receptors

### CXCR4 Expression and Function

Originally known as leukocyte-derived seven-transmembrane domain receptor (LESTR) or Fusin, CXCR4 was first described as an orphan GPCR that facilitates HIV-1 fusion with target cells—hence the name “Fusin” (46). CXCL12 is the unique and specific chemokine for CXCR4 (47). Its binding promotes the activation of heterotrimeric Gα*βγ* proteins, and the subsequent activation of multiple signaling pathways controlling calcium mobilization, actin polymerization, cytoskeletal rearrangements, gene transcription, and receptor internalization (48–51), cell proliferation, cell survival, and even apoptosis (52–55).

CXCR4 is an homeostatic receptor that is widely expressed both in embryonic and in adult tissues (1). As previously indicated, data from *Cxcr4*-deficient mice correlate with those observed in *Cxcl12*-knockout mice (29–31, 56), showing defects in hematopoiesis and in nervous and cardiovascular development. The importance of the CXCR4/CXCL12 axis is reflected not only by the fact that both knockout mice are embryonic lethal, but also because the ligand and the receptor are highly conserved members of chemokine and chemokine receptor families during evolution (57).

CXCR4 is ubiquitously expressed in the hematopoietic system, although its expression level is quite variable among cell types. As with other chemokine receptors, CXCR4 is important in leukocyte trafficking and arrest in specific niches, both under homeostatic and pathological conditions, by recruiting cells to sites of inflammation. During antigen-presenting cell/T cell contact, CXCR4, among other chemokine receptors, is recruited to the pSMAC (peripheral SupraMolecular Activation Cluster) where it contributes to the integrin activation needed to generate a productive immunological synapse and proper T cell activation (25). CXCL12/CXCR4 are thus key elements for the adaptive and innate immune response and also for BM organization and maintenance (58). Indeed, CXCR4 and CXCL12 are largely responsible for hematopoietic stem cell migration (59), homing (60–63), and survival (64, 65) in BM.

CXCR4 is also expressed in several non-hematopoietic tissues, such as lung, liver, kidney, gastrointestinal tract, adrenal gland, ovary, and brain. The importance of CXCR4 in adult tissues has been demonstrated using conditional *Cxcr4*-knockout mice, which have served to assign an important role of CXCR4 in regulating central nervous system development (66), and vasculature development in the gastrointestinal tract (30) and the kidney (67).

CXCR4 and CCR5, are the primary co-receptors for HIV-1 entry into target cells. Indeed, HIV strains are classified as X4- or T-tropic and R5- or M-tropic depending on the chemokine receptor used for cell infection (68). In newly infected individuals HIV-1 entry occurs mainly through CCR5 and CD4, they are M-tropic R5 strains which predominate in the acute and asymptomatic phases of HIV infection. CD4^+^ T helper type 1 (Th1) cells, which express high levels of CCR5 (69, 70), are implicated in maintaining the asymptomatic status (71, 72). The viral use of CXCR4 emerges later in disease and correlates with the phase of immunological deficiency and AIDS progression (73, 74).

Inherited heterozygous autosomal dominant mutations in the *CXCR4* gene cause WHIM syndrome (75, 76), a severe combined immunodeficiency disease characterized by susceptibility to human papilloma virus infection, which causes warts, condyloma acuminata and carcinomas. These patients can suffer neutropenia, B cell lymphopenia, hypogammaglobulinemia which is related to recurrent infections and BM myelokathexis characterized by myeloid hyperplasia and increased numbers of mature, senescence neutrophils in the bone marrow (75). The mutations in *CXCR4* result in a stop codon that eliminates the last 10–19 amino acids at the C-terminus, or alter specific key residues for receptor phosphorylation in this domain. In all cases, mutations impair CXCR4 internalization (48, 77), sustaining its activity and enhancing G protein- and β-arrestin-dependent signaling.

While considered a homeostatic receptor, the expression of CXCR4 can be modulated by different pathological conditions. For example, CXCR4 is overexpressed by many tumor types, including breast (34), ovarian (78), prostate (79), melanoma (80), and neuroblastoma (81), among others. Also, the elevated expression of CXCR4 in metastatic lesions correlates with tumor progression and with preferential metastatic sites of the primary tumor (82–84). Studies in mice show that CXCR4 is a good target in cancer as its blockade impairs the spread of cancer cells and metastasis in several cancer models (34, 85, 86). The CXCL12/CXCR4 axis is also involved in tumor growth, tumor cell interactions with the microenvironment (87), vasculogenesis and angiogenesis (88). In this setting, hypoxia has been related to the upregulation of CXCR4 expression, suggesting that this receptor is involved in tumor progression (89, 90).

Inflammation has also been identified as a relevant factor for CXCR4 modulation, as TGF-β1 (91), VGEF (90), and bFGF (92) are reported to upregulate CXCR4 expression, whereas other cytokines such as IL-5 (93), IFNα and IFNγ (94) downregulate its expression. Overall, these data illustrate the involvement of the CXCR4/CXCL12 axis in the development and progression of immunodeficiency and inflammatory diseases and cancer, and underline its interest as a target for therapeutic intervention.

### ACKR3 Expression and Function

ACKR3, also known as RDC1 and CXCR7, was first identified as an orphan GPCR, and was later described as a high-affinity receptor for CXCL12 and CXCL11 (44, 95). Instead of having the canonical DRYLAIV motif, which is involved in coupling to G proteins, it contains the sequence DRYLSIT (96) and is, accordingly, included in the group of “atypical chemokine receptors” (ACKR)—hence the name “ACKR3.” It acts as a scavenger receptor for CXCL12 (97, 98), but also triggers the β-arrestin pathway (45), and can be also implicated in modulating CXCR4 functions by forming heterodimers (99, 100).

In adults and under homeostatic conditions, ACKR3 is not expressed at the cell surface of human or mouse leukocytes in peripheral blood (101), but is expressed in B cells and dendritic cells in secondary lymphoid organs (102). ACKR3 is also expressed in several non-hematopoietic cells, for example, in endothelial and mesenchymal cells and in neurons. Reflecting its importance in embryonic tissues, *Ackr3*-knockout mice die perinatally and show severe defects in cardiovascular, kidney, and brain development, but not in hematopoiesis (100).

In the presence and absence of ligands, ACKR3 continuously recycles between the cell membrane and the endosomal compartment. Ligands trigger a moderate increase of ACKR3 internalization which concours with a marked uptake and degradation of CXCL11 and CXCL12, thus supporting its scavenging role (98). As also occurs for CXCR4, ACKR3 surface expression is increased in pathological situations, both in leukocytes and endothelial cells. In a prostate cancer model, CXCL8 increases the expression of mRNA and protein levels of ACKR3 (103); IL-1β triggers ACKR3 expression in HUVEC cells (104); and other external factors such as lipopolysaccharides associate with ACKR3 upregulation in the pulmonary epithelium modulating microvascular permeability during acute pulmonary inflammation (105). Although the mechanisms are not fully elucidated, *in vitro* and *in vivo* studies have described that higher levels of ACKR3 correlate with increased cell proliferation and invasive migration, that is with tumor growth and metastasis (106–108).

Similar to CXCR4, ACKR3 is implicated in some autoimmune diseases, such as rheumatoid arthritis (104), inflammatory bowel disease (109), and experimental autoimmune encephalomyelitis (EAE) (110, 111).

## CXCR4/CXCL12 Structure

While the sequence identity among chemokines varies greatly (15–90%), there is a high level of conservation in their tertiary structures, as revealed by nuclear magnetic resonance and X-ray crystallography (16). The structure of CXCL12 was first described in 1997 using nuclear magnetic resonance spectroscopy (112). Its 67 residues present a typical chemokine folding with an eight residue-long N-terminus followed by a central body assembled by three antiparallel β-strands forming a β-sheet covered by an α-helix. CXCL12 presents a high percentage of basic residues dispersed along the N-terminus and the main body that are important for the binding to specific residues in the receptor. A two-step binding site model has been proposed (112, 113) for the interaction between CXCL12 and CXCR4, with a first interaction of the central body of the chemokine with the N-terminus of CXCR4 in the Chemokine Recognition Site 1 (CRS1), allowing the ligand to obtain an optimal orientation on the receptor, which enables the N-terminus to penetrate deep into the receptor and bind to the CRS2 region. This structure may shift between monomers and dimers depending on the solution conditions, a fact that might affect CXCL12 activity. In addition, a non-dissociating CXCL12 dimer that binds CXCR4, induces Ca^2+^ mobilization, but does not mediate *in vitro* cell migration has been described (114), suggesting specific functions associated with distinct ligand conformations.

The first high-resolution structure of CXCR4 was published in 2010 (115), with five independent receptor structures reported in complex with two antagonists, the small molecule IT1t and the cyclic peptide CVX15; thus, all the structures presented an inactive state conformation. As described for other GPCRs (116, 117), CXCR4 displays the canonical 7-helix bundle arrangement crossing the membrane, linked by three extracellular loops (ECL1–3) and three intracellular loops (ICL1–3). Also, it contains a long N-terminus composed of 34 residues and a C-terminus where the VIII typical helix was not clearly defined. A more recent structure of CXCR4 has been reported in complex with the viral chemokine vMIP-II (118), with very similar features to those described previously.

**Figure 1:**
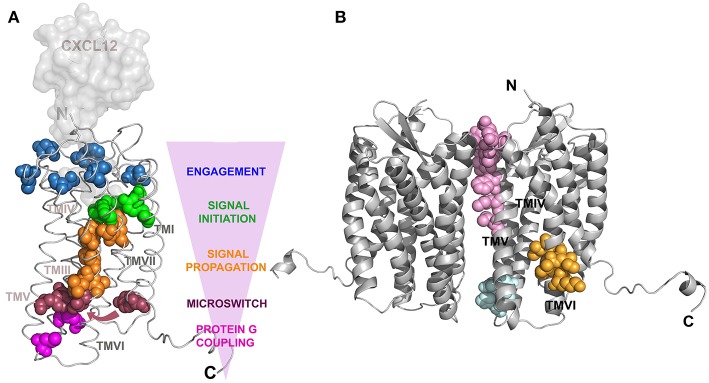
Structure of CXCR4. Models created using the Swiss-Model web server (119), with PDB 3oe8 as a reference for CXCR4 and PDB 1a15 for CXCL12, which was superposed to vMIP-II present in PDB 4 rws to build a CXCR4:CXCL12 complex. **(A)** Ribbon representation of a CXCR4 monomer in complex with a surface representation of CXCL12. Residues implicated in CXCL12 signal transmission are depicted as colored spheres corresponding to the specific state of signal transference showed by the pink arrowhead indicating the direction of the CXCR4:CXCL12 axis signal transit. Blue colored residues are part of the CXCL12 binding site that contact the green colored residues responsible for the initiation of the signal transmission toward the cytoplasm. Subsequent signal propagation through a hydrophobic bridge, in orange, allows conformational changes in TMVII inducing translation of specific residues (red spheres), depicted by an arrow, that will act like a microswitch and position the G-protein binding residues (pink) in an active conformation. [Adapted from Wescott et. al. (51)]. **(B)** Cartoon representation of the CXCR4 dimer. Residues contacting between monomers are shown by pink spheres for CXCR4:IT1t complex, cyan for CXCR4:CVX15 complex and orange for oligomerization inhibition after mutating residues 239–246 in TMVI (21).

CXCL12 binding to CXCR4 promotes conformational changes in the transmembrane domains governed by a chain of “signaling” residues present along the transmembrane α-helices identified by mutagenesis studies [(51); Figure 1A]. CXCL12 binding to the CRS2 region facilitates the interaction of the first two N-terminal residues of the ligand, K and P, with a group of residues mainly present at this domain in the receptor to engage and trigger signaling [Figure 1A, blue and green residues; (112)]. Next, eight residues present in the transmembrane (TM) segments TMVI and TMVII (Figure 1A, orange residues), which form a continuous rod through the receptor, connect the signal initiation with the next residues (Figure 1A, red). This connection is critical for signaling since these residues are components of the microswitch allowing G protein coupling. Of note, residues F248 to V242 in TMVI are in contact with almost all the conserved motifs present in the GPCRs that are crucial for signaling, including motifs CWxP in TMVI, NPxxY in TMVII, DRY in TMIII, and Y(x)_5_KL in TMV. This assigns a role for these residues in controlling the transition between active and inactive states, by enabling helix and side chain translations, as described using mutational studies of this hydrophobic bridge in different receptors (120–122).

The ability of GPCRs to homo- and heterodimerize has been described (123) and the crystallographic structures of CXCR4 highlight the regions implicated in these interactions (115, 118). The implicated residues are located mainly at the extracellular portion of helices V and VI in the case of the CXCR4:IT1t complex (Figure 1B, pink residues) and at the base of helices III and IV in the CXCR4:CVX15 complex (Figure 1B, cyan residues). These residues differ from those previously described in models of GPCR dimerization, where contacting residues were allocated in helices I and IV (124, 125). The functional implications of these differences for the CXCR4 life cycle remain unclear, but since there is a low sequence identity in the dimerization region among dimerizing GPCRs, it might be a feature specific to CXCR4. A novel region for allosteric regulation of CXCR4 oligomerization has also been described (21). Residues located at the N-terminus of TMVI (Figure 1B, orange residues) were mutated and the receptor remained able to dimerize but not to form large nanoclusters after ligand binding. This mutant receptor was able to trigger some calcium flux and to promote cell adhesion but could not trigger cell migration, suggesting that receptor nanoclustering is essential for complete CXCR4-mediated functions.

## CXCL12-mediated Signaling Pathways

CXCR4 exists in the plasma membrane as a monomer, dimer, and higher-order oligomers. It can also interact with other chemokine receptors (i.e., ACKR3, CCR5, and CCR2) and other cell surface proteins such as the TCR, CD4, tetraspanins, and other GPCRs (21, 50). This wide variety of interactions predicts a high signaling potential and diversity for CXCR4.

Like most GPCRs, CXCR4 was initially believed to act as monomer. Resonance energy transfer techniques revealed that CXCR4, as with other chemokine receptors, forms homo- and heterodimers (21), even in the absence of ligand stimulation. Defining the contribution of different receptor conformations to functional response is a challenging endeavor. Recent studies using advanced microscopy techniques indicate that CXCR4 and also CCR7 form ligand-induced higher-order oligomers that are critical for receptor function (21, 126). Nevertheless, ligand-induced oligomerization is solely capable of mediating signaling, as blocking the formation of CXCR4 oligomers diminishes the functional response (21). Overall, these data indicate that CXCR4 exists in multiple conformations and highlight the importance of receptor oligomers for full receptor function. While binding experiments at equilibrium showed negative cooperativity between the subunits of a receptor dimer, supporting high-affinity binding of only a single chemokine molecule per receptor dimer (127), the stoichiometry of these receptor conformations with the ligands is still unclear.

As occurs with other GPCRs and their ligands, CXCL12 engagement with CXCR4 triggers conformational changes in the latter promoting the dissociation of GTP-loaded heterotrimeric G proteins (16); the GTP-loaded Gα subunit and the Gβγ heterodimer then activate downstream effectors. For CXCR4, as for chemokine receptors in general, signaling is abolished by pertussis toxin, indicating the involvement of Gα_i_ in the signal cascade (128). However, chemokine receptors can be also coupled to Gα_q_ or Gα_11_ in a cell- and stimulus-dependent manner (129–131).

Gα_i_ inhibits adenylyl cyclase, promoting a reduction of cAMP and activating Src-related kinases, MAPK, PI3K, small GTPases and PLC-β (phospholipase C). In turn, PLC-β activation generates the second messengers diacylglycerol (DAG) and inositol 1,4,5-triphosphate (IP_3_), which are required for cell proliferation and migration. Some reports indicate that Gβγ also activates PLC-β (50, 132). Gα_i_ also triggers biphasic PI3K activation, a pathway related to cell migration, proliferation, and survival (133). PI3K can also be activated directly by the Gβγ dimer (134), and PI3K catalytic subunits have a Ras-binding domain, which can be recognized by small GTPases (such as Ras). Both, the PI3K signaling cascade, which includes Akt and mTOR proteins, and the MAPK pathway, have important roles in actin reorganization and cell migration (53, 135, 136).

Other mechanisms activated by CXCR4/CXCL12 are G protein-independent, i.e., activation of GRK family proteins. It is known that GRK2, GRK3, and GRK6, and also PKC, are implicated in phosphorylation of serine/threonine residues in the CXCR4 C-terminus, which contributes to CXCR4 desensitization (137, 138). The phosphorylated residues of the CXCR4 C-tail trigger β-arrestin recruitment, thereby simultaneously promoting the uncoupling of G proteins (139), and resulting in β-arrestin-dependent receptor endocytosis.

Different regulatory mechanisms for CXCR4 have been also described (49). CXCL12 triggers a rapid ubiquitination and degradation of CXCR4 (140). The process requires a GRK6-mediated phosphorylation of several serine residues at the C terminal end of CXCR4 (141) and receptor internalization (142). Other regulatory mechanisms involve the ability of CXCR4 to associate with other receptors, and a role for ACKR3/CXCR4 heterodimers regulating CXCL12-mediated G protein signaling has been described (99). Indeed, in glioblastoma cells, ACKR3/CXCR4 crosstalk affects major signaling pathways related to cell survival, proliferation, and migration (143).

CXCR4 also interact with other chemokine receptors, with different functional consequences—as illustrated by its interaction with ACKR3. CXCR4 can form heterodimers with CCR5, and together with the TCR of CD4^+^ cells, modulates HIV infection (23, 144). Examples of other non-chemokine receptors known to interact with CXCR4 include the cannabinoid receptor CB2, with implications for tumor progression (145). Also, the delta opioid receptor forms heterodimers with CXCR4 and might modulate pathological processes (22).

## CXCL12/CXCR4/ACKR3 in Autoimmune Diseases

Autoimmune diseases result from pathological immune responses against self-antigens. The clinical manifestations are initiated after tissue infiltration of immune cells, which trigger the uncontrolled attack. Because of its central role in the control of immune cell trafficking, the chemokines, produced by stromal cells, tissue cells, and activated cells of the innate immune system, are essential molecules in the development of autoimmune responses. Although CXCL12 was originally classified as a homeostatic chemokine, it also plays an important role during inflammation (see Table 1). Here, we review the involvement of the CXCL12/CXCR4/ACKR3 axis in some of the most prevalent autoimmune diseases: psoriasis (146), multiple sclerosis (161), rheumatoid arthritis (162), lupus (155), type I diabetes (T1D) (163), and inflammatory bowel disease (164, 165).

**Table 1:** Summary of CXCL12-mediated effects on cells in autoimmunity.

**Disease**	**Target cells**	**Effects**	**References**
Psoriasis	Macrophages	Cell migration	(146)
	Lymphocytes	Cell migration	(146)
	Endothelial cells	Angiogenesis	(146)
	Keratinocytes	Proliferation	(147)
Multiple sclerosis	Mononuclear cells	Limits cell migration	(148)
	Regulatory T cells	Polarization	(148)
Rheumatoid arthritis	Leukocytes	Cell migration retention in inflamed areas	(91, 149)
	Endothelial cells	Angiogenesis	(150)
	Chondrocytes	Necrosis release of proteases	(151, 152)
	Osteoclasts	Attraction, differentiation, activation resistance to apoptosis	(153, 154)
Lupus	Leukocytes	Cell migration	(155)
	Epithelial cells	Cell migration	(156)
	Treg cells	retention in bone marrow	(157)
	T cells	Chemorepulsive favors autorreactive T cell balance	(158)
	β-cells	Differentiation, anti-apoptotic protection	(54, 159)
	Endothelial cells	Angiogenesis	(160)
	Progenitors	Cell recruitment	(160)
IBD	T cells	Cell migration	(109)

### Psoriasis

Psoriasis is a chronic inflammatory disease affecting the skin. It affects 2 to 3% of the population worldwide (166). It is characterized by epidermal thickening linked to enhanced proliferation and aberrant terminal differentiation of epidermal keratinocytes. It also shows accumulation of inflammatory leukocytes—in particular dendritic cells, macrophages, and T cells (167)—and pronounced inflammatory angiogenesis, leading to vascular remodeling (168). Immune cell infiltration predicts an important role of chemokines in psoriasis as well as in other inflammatory skin diseases such as atopic dermatitis and mastocytosis. Indeed, comparative gene expression profiling showed significantly higher expression of *CCL4, CCL20, CXCL8*, and *CXCL2* in skin lesions of patients with psoriasis as compared with atopic dermatitis, and the opposite was found for *CCL13, CCL18*, and *CCL27*, reflecting the distinct infiltrating cell types in the two conditions (169, 170). Especially relevant is the expression of CX3CL1 (fractalkine), whose receptor CX3CR1 has been identified as a psoriasis susceptibility gene (171), and is known to attract specific subtypes of T cells and immature mast cells to the inflamed skin (172).

The presence of angiogenesis in psoriatic lesions has led to a focus on chemokines with angiogenic properties, in particular CXCL12 (173), which is expressed in the skin (174) and is upregulated by the angiogenic factor VEGF-A (175). Elevated mRNA levels of both *CXCL12* and *CXCR4* have been found in lesions of psoriatic skin (176). In a model of imiquimod-induced skin inflammation in VEGF-A transgenic mice, blockade of CXCR4 inhibited skin inflammation, and was associated with reduced angiogenesis and inflammatory cell accumulation, including dermal CD4^+^ T cells, intra-epidermal CD8^+^ T cells and macrophages (146). Other reports have shown a beneficial role of the CXCL12/CXCR4 axis in psoriasis. For example, in a mouse model of IL-23-induced psoriasiform dermatitis, specific depletion of *Cxcr4* in keratinocytes led to increased keratinocyte proliferation and enhanced the effects of proliferative Th17 cytokines (147). Psoriasis is nonetheless a human-specific disease and therefore most experimental mouse models do not fully summarize all the characteristics present in humans.

### Multiple Sclerosis

Multiple sclerosis (MS) is the most common autoimmune disease affecting the central nervous system (CNS). It is characterized by chronic inflammation, demyelination, and neurodegeneration (177), triggering CNS dysfunction, visual disorders, and motor deficits. The prevalence of MS varies with country, but it is higher in Europe and North America (178). As in other autoimmune diseases, MS is a multifactorial disease caused by a combination of different factors, genetic predisposition, environment, viral infections that activate the immune system, and an uncontrolled autoimmune inflammation (179), but the pathogenesis is still not well-understood. Cell extravasation into the brain parenchyma is needed to provoke tissue damage; however, the existence of the blood brain barrier and the blood-cerebrospinal fluid (CSF) barrier makes the pathogenesis of CNS disease different from other inflammatory diseases (180). Immune cell activation occurs primarily in cervical lymph nodes (181), but autoimmune T cells require myeloid cell-mediated re-stimulation in the CNS to survive and trigger the disease (169, 182–184). The encounter between activated T cells and antigen-loaded myeloid cells along the abluminal surfaces of subpial vessels confers competence to T cells to invade the parenchyma and to mediate disease (184).

Chemokines, their receptors and adhesion molecules orchestrate leukocyte trafficking in the CNS (170). *Ccr6*-deficient mice did not develop experimental allergic encephalitis (EAE) although *Ccr6*-deficient T cells were efficiently primed *in vivo*, and their effector functions were unaffected (185). Studies have identified P-selectin, α4 integrins and CCR6 as the promoters of lymphocyte migration into the CSF (186). Accordingly, the ligand for CCR6, CCL20, is constitutively expressed by epithelial cells of the choroid plexus in mice and humans, indicating that T cells must cross the blood-CSF barrier to initiate EAE (181). However, a significant albeit minor fraction of cerebrospinal T cells in healthy humans express CCR6, suggesting that additional molecules are needed to support this trafficking (169).

CXCL12 is constitutively expressed in the adult CNS, and is rapidly up-regulated under some pathological situations such as HIV-1-associated dementia, brain tumor, or neuroinflammation (187–193). Due to the local hypoxia, during ischemia, the transcription factor hypoxia-inducible factor-1 (HIF-1), which regulates CXCL12 gene expression in endothelial cells is activated (194). CXCL12 is detected in the CSF of patients with MS (161, 195, 196), and its levels are high on astrocytes in active lesions, throughout the lesion areas as well as on some monocytes/macrophages within vessels (196, 197). During the induction of EAE, CXCR4 blockade promotes loss of the typical intense perivascular cuffs, which is then replaced with mononuclear cell infiltration of white matter (198). This observation concours with the increased levels of IFNγ and other cytokines associated with monocyte and microglial activation, demyelination, and with stronger clinical severity of the disease (199). The available evidence thus indicates that CXCL12 restricts the intraparenchymal migration of mononuclear cells during autoimmune disease at the CNS. A molecule derived from CXCL12, P2G-CXCL12, diminished the progression of EAE (200). A similar effect was obtained using another variant of the N-terminal domain of CXCL12, obtained as a result of phage display, LGGG-CXCL12 (201).

In EAE, ACKR3 expression increases at the infiltration sites on endothelial barriers, indicating that its scavenger role on CXCL12 is essential for leukocyte entry into the CNS parenchyma (111). A specific antagonist of ACKR3, CCX771, prevents immune cell infiltration into the CNS improving the clinical signs of EAE and preserves axonal integrity (202). ACKR3 has also been implicated in migration of activated microglia cells, an effect linked to amelioration of clinical severity (203).

### Rheumatoid Arthritis

Rheumatoid arthritis (RA) is one of the most common autoimmune disorders and affects 1% of the population (204). It is characterized by persistent inflammation driven by proliferating fibroblasts in the synovial tissue, as well as T and B cell, neutrophil, and monocyte trafficking into the joint (205, 206). Cells invading RA pannus lesions express proinflammatory cytokines, chemokines, and matrix metalloproteinases that contribute to progressive cartilage and bone destruction (207). The RA pannus also shows strong neovascularization (208), particularly in the early stage of the disease (209), which might facilitate cell extravasation into the synovium. The etiopathology of RA has been difficult to define; however, as in other autoimmune processes, genetic predisposition, environmental factors, and an uncontrolled immune response are clearly implicated (206).

Chronically inflamed non-lymphoid tissues can host highly organized aggregates of ectopic lymphoid structures. These structures usually appear during the course of persistent infections, transplant rejection, cancer and autoimmune diseases such RA (210). The process occurs in response to mediators of inflammation such as chemokines, cytokines, and bioactive lipids produced by tissue-resident cells, and is able to regulate the recruitment and organization of lymphocytes.

Synovial fibroblasts and monocytes/macrophages are the main source of the inflammatory chemokines expressed in the inflamed RA joints (211, 212), of which CXCL8, CXCL5, CXCL10, CCL5, and CCL20 are primarily involved in the selective recruitment of immune cells. Especially significant to this process is CXCL8, which is released by osteoclasts in response to anti-citrullinated peptides antibodies, and attracts neutrophils, leading to bone erosion and pain (213). By acting in an autocrine or paracrine manner, the chemokines also activate fibroblasts in the RA synovium (214).

Synovial fibroblasts and endothelial cells also express CXCL12 (149), and the local presence of IL-15 and TGFβ enhances CXCR4 expression by immune cells (91). These observations support the hypothesis that the CXCL12/CXCR4 axis retains activated immune cells in the inflamed joint, thus contributing to the chronic inflammation. Indeed, CXCL12 expression correlates with bone erosion (215). CXCL12 attracts osteoclast precursors and stimulates their differentiation and bone-resorbing activity, upregulates the production of some metalloproteases, and confers resistance to apoptosis in osteoclasts (153, 216). In addition, *in vitro* experiments demonstrate that CXCL12 promotes chondrocytes necrosis, suggesting a role of this chemokine in cartilage damage.

CXCL12 has also been implicated in synovial neovascularization (91). As in tumors, the presence of hypoxia in inflamed joints activates HIF-1, inducing VEGF and CXCR4 expression (90). In addition, ACKR3 has been associated with angiogenic processes in RA synovium (104), although its role in the pathogenesis of RA has been less studied.

### Systemic Lupus Erythematosus

Systemic lupus erythematosus (SLE) is a multisystem autoimmune disease characterized by circulating autoantibody-autoantigen complexes and infiltration of different types of leukocytes, promoting inflammation, and organ damage (217). Lupus nephritis is a major cause of morbidity and mortality in up to 60% of patients with SLE and is characterized by kidney inflammation (218). While the precise etiological mechanisms of SLE are unknown, genetic, hormonal, and environmental factors, as well as immune abnormalities, have been identified (219). Studies in animal models, also corroborated in humans point to essential roles of chemokines, including CXCL13, CXCL12, CXCL9, CXCL10, among others (220).

In several lupus models, CXCR4 expression is increased in B cells, plasma cells, T cells, neutrophils, and monocytes (221), which concurs with the increased CXCL12 expression in the kidney (222). CXCL12 also affects renal tissue cells. In glomerulonephritis, CXCR4 is overexpressed in parietal epithelial cells of the kidney, triggering their migration into the glomerular tuft where they form hyperplastic lesions (156). Administration of anti-CXCL12 neutralizing antibodies in NZB/WF_1_ mice increases their survival rate, and reduces IgG deposition and renal inflammation (222). In patients with lupus nephritis, CXCL12 expression is increased in the tubules and glomeruli of kidneys (223), and an accumulation of B cells is found in renal biopsies (224). In accordance, CXCR4 overexpression on B cells from these patients positively correlates with disease activity and kidney involvement (225).

### Type I Diabetes

Type I diabetes (T1D) is an autoimmune disease caused by the selective destruction of insulin-producing pancreatic β-cells following infiltration of the islets of Langerhans by immune cells (insulitis) (226). This is accompanied by the progressive loss of glucose homeostasis that induces the classical early symptoms of the disease, polydipsia, polyphagia, and polyuria (211). As in other autoimmune diseases, genetic predisposition, environmental factors, and acute and/or chronic inflammation caused by acute and/or persistent pathogen infection are known factors in the development of T1D (212). Cardiovascular events, including myocardial infarction, stroke, angina, but also microvascular complications such as retinopathy, nephropathy, and neuropathy, all associate with T1D, and cardiovascular disease is the primary cause of death in patients (212).

Multiple chemokines have been associated with T1D and/or its complications. In particular, CXCL10 was identified as the central chemokine expressed in the islet environment of prediabetic animals and in patients with T1D (227), whereas CCL5, CCL8, CXCL9, and CX3CL1 are also present but at low levels (228). The role of chemokines is believed to be related to immune cell infiltration into the β-islets, which requires the coordinated action of many proteins involved in cell movement.

Blockade of CXCL12 inhibits insulitis and diabetes development (157, 163). It was proposed that the retention of Treg cells in the BM by CXCL12 alters the balance of T cell subpopulations in favor of autoreactive T cells. However, an opposite effect of CXCL12 inhibition was also reported, showing that a population of T cells attracted by CXCL12 protects recipient mice from the capacity of diabetogenic T cells to transfer diabetes (229). CXCL12 is also chemorepulsive on diabetogenic T cells, while mediating firm adhesion of normal T cells (158) and the retention of Treg cells (230). Pancreatic lymph nodes of NOD mice have reduced levels of Treg cells and this associates with decreased expression of CXCL12, whereas the recovery of normal levels of glucose in blood associates with the restoration of the Treg population in peripheral LNs (231). In addition, CXCL12/CXCR4 signaling is important for β-cell differentiation and the genesis of pancreatic islet (54). It also exerts anti-apoptotic and anti-necrotic effects on β-cells to protect from diabetogenic agents (159).

The function of CXCL12 as a proangiogenic factor together with its ability to recruit endothelial progenitor cells accelerates wound healing in diabetes (232, 233). But the same effects can promote the progression of retinopathy associated with T1D. Tissue ischemia and reduced blood flow stimulate aberrant neovascularization and normal retinal architecture damage, causing impaired vision. CXCL12 expression is controlled by HIF-1 (194), and its levels increase as diabetic retinopathy progresses, contributing to angiogenesis and recruitment of endothelial progenitor cells to the site of vascular injury (160). Similarly, during acute renal failure, CXCL12 expression in the kidney increases triggering progenitor cells homing to the injured kidney (234), a process that might also occur during the diabetic nephropathy.

### Inflammatory Bowel Disease

Idiopathic Inflammatory Bowel Diseases (IBDs) comprise two chronic intestinal diseases, ulcerative colitis and Crohn's disease, triggered by a dysregulated immune system response to environmental in genetically susceptible individuals (235, 236). Evidence suggests that the disease is a consequence of the inappropriate control of the immune response against enteric microbiota, which promotes the influx of inflammatory cells directed by chemokines (237–239).

Both CXCL12 and CXCR4 are expressed by intestinal epithelial cells (240, 241) and CXCR4 is also expressed in cells of the lamina propria (242, 243). Both are upregulated in IBD patients (240, 243) and participate in T cell-recruitment, mainly of memory Th1 cells (109). Some polymorphisms of the CXCL12/CXCR4 axis have associated with IBD progression and disease severity (244). Administration of a CXCR4 antagonist ameliorated colonic inflammation in a dextran sulfate sodium-induced model of colitis. The same study showed that the CXCR4 antagonist decreased TNFα and IFNγ production by mesenteric lymph node cells, whereas IL-10 production was unaffected. The authors also indicated that these antagonists do not affect the percentage of mesenteric Foxp3^+^CD25^+^ T cells (164). Later, using the same murine model of colitis, it was demonstrated that AMD3100 also enhanced epithelial barrier integrity (245). A recent report has suggested a role of CXCR4 in mesenchymal stem cells (MSCs) homing to the inflamed intestinal tissues (246). MSCs play an important role in tissue repair and regeneration but also have immunoregulatory properties that might contribute to reduce inflammation (247). ACKR3 is also expressed in peripheral T cells as well as in those of the lamina propria, but only peripheral T cells show ACKR3 upregulation during IBD (248). Regarding the ability of ACKR3 to form heterodimers with CXCR4 and to modulate its function, it is possible that ACKR3 upregulation allows a rapid increase of the influx of T cells to inflamed mucosa. Nonetheless, a direct role of ACKR3 in modulating CXCL12 levels (249) or even in modulating cell survival should not be discarded (250), although this observation needs be confirmed.

## Summary/Perspectives

CXCL12 is a ubiquitous chemokine whose expression is regulated by circadian signals (251) and inflammatory mediators. Based on its essential role in embryogenesis and in homeostasis, it has been traditionally classified as a homeostatic chemokine. CXCL12 and CXCR4 are essential during hematopoiesis, as they play an important role in hematopoietic stem cell retention in BM and in facilitating B cell differentiation. Plerixafor, a CXCR4 antagonist, is currently in use to mobilize hematopoietic stem and progenitor cells for autologous transplantation, and to increase the efficacy of chemotherapeutic agents in several hematological malignancies. CXCR4 and ACKR3 are also important for tumor growth and dissemination. Accordingly, CXCR4 antagonists, which impair tumor dissemination, are in clinical trials to evaluate their use in combined therapies with anti-cancer treatments. Many inflammatory and autoimmune diseases show increased expression of CXCL12, suggesting that it could also be considered an inflammatory chemokine or at least forms part of a mechanism to maintain the homeostatic balance in diseased tissues. Indeed, administration of CXCL12 antagonists delays inflammatory disease onset and/or retards disease progression.

CXCL12 and its cognate receptors are a paradigm of cell-to-cell signaling; however, their biology is more complex than originally anticipated. CXCL12 can associate with other chemokines, as well as with other inflammatory mediators that modulate its functionality, and is also a target for post-transductional modifications such as truncation, nitration or citrullination processes that alter its responses. Also, chemokine receptors adopt different conformations at the cell membrane: CXCR4 is present at the cell surface as monomers, dimers (both homo- and heterodimers), and oligomers. These conformations increase cell plasticity, and shape the responses to the biological environment; for example, by forming heterodimers, ACKR3 modulates CXCR4-mediated functions, thereby contributing to the functional plasticity of CXCL12.

Overall, the data outlined in this review reflect the relevance of CXCL12 in homeostasis as well as in several diseases such as cancer or AIDS where its role is well-known, but also highlight its participation in autoimmune diseases. There, CXCL12 shows several functions, mediates cell infiltration and retention in specific tissues, triggers angiogenesis and even, in some cases, promotes cell proliferation. These effects are sometimes essential for the development of the disease or for increasing its severity and therefore justify the search of new antagonists. Compounds that abolish CXCL12 binding or partially affect some of its functions without altering others, should be incorporated in the clinical armamentarium to treat these prevalent diseases.

## Author Contributions

EG-C, CS, JV-D, YJ, JR-F, and MM wrote the manuscript. CS and JV-D performed the figures. MM completed the table and coordinated the manuscript. EG-C and JR-F coordinated the bibliography and the format of the manuscript.

### Conflict of Interest Statement

The authors declare that the research was conducted in the absence of any commercial or financial relationships that could be construed as a potential conflict of interest.
